# Forward flight stability in a drone-fly

**DOI:** 10.1038/s41598-020-58762-5

**Published:** 2020-02-06

**Authors:** Hao Jie Zhu, Xue Guang Meng, Mao Sun

**Affiliations:** 1 Ministry-of-Education Key Laboratory of Fluid Mechanics, School of Aeronautic and Engineering, Beihang University, Beijing, 100191 China; 2 State Key Laboratory for Strength and Vibration of Mechanical Structures, School of Aerospace, Xi’an Jiaotong University, Xi’an, 710049 China

**Keywords:** Computational biophysics, Fluid dynamics

## Abstract

Previous studies on forward flight stability in insects are for low to medium flight-speeds. In the present work, we investigated the stability problem for the full range of flight speeds (0–8.6 m/s) of a drone-fly. Our results show the following: The longitudinal derivatives due to the lateral motion are approximately 3 orders of magnitude smaller than the other longitudinal derivatives. Thus, we can decouple these two motions of the insect, as commonly done for a conventional airplane. At hovering flight, the motion of the dronefly is weakly unstable owing to two unstable natural modes of motion, a longitudinal one and a lateral one. At low (1.6 m/s) and medium (3.1 m/s) flight-speeds, the unstable modes become even weaker and the flight is approximately neutral. At high flight-speeds (4.6 m/s, 6.9 m/s and 8.6 m/s), the flight becomes more and more unstable due to an unstable longitudinal mode. At the highest flight speed, 8.6 m/s, the instability is so strong that the time constant representing the growth rate of the instability (disturbance-doubling time) is only 10.1 ms, which is close to the sensory reaction time of a fly (approximately 11 ms). This indicates that strong instability may play a role in limiting the flight speed of the insect.

## Introduction

When the lift balances the weight, the thrust balances the drag and the moments about the center of mass are zero, an insect is in equilibrium flight. Dynamic stability answers the question that when the flight is disturbed, whether or not the insect returns to its equilibrium state without applying active control. Knowing whether or not the flight is stable and if it is unstable, how fast the instability grows, is essential to understanding the controllability and maneuverability of the insect. It also is of great importance to the study of its internal control systems, e.g. sensory systems and motor systems. Besides, understanding the flight stability of insects can be helpful in designing insect-like flying robots. As a result, in the last fifteen years or so, many studies have been conducted in this area^1–14^. However, most of these previous works were for hovering flight, while very few were on forward flight. As far as we know, only three studies were on forward flight: Taylor and Thomas^1^ investigated the stability in forward-flying locusts at medium flight speed. Only longitudinal motion was considered. In the study, the aerodynamic derivatives were measured and the insect was tethered in the experiment. They obtained an unstable divergence mode and two stable modes (a subsidence one and an oscillatory one, respectively). Xiong and Sun^13^ investigated the longitudinal flight dynamics in forward flight in a bumblebee. Xu and Sun^14^ considered the lateral flight dynamics of the same insect. Flight dynamics at hover and at speeds 1 m/s, 2.5 m/s, 3.5 m/s and 4.5 m/s was studied. Natural modes of motion at each flight speed were identified. Morphologic parameters and flight data of the insect were taken from literature^15^. Hovering flight and low speed flight have similar model structure: three longitudinal modes, one of them being a weakly unstable oscillatory mode and the same number of lateral modes, one of them being weakly unstable. The flight is unstable owing to the two modes of instability. As speed increases, the model structure changes and the flight is approximately neutrally stable at speeds 2.5 m/s and 3.5 m/s and becomes unstable again at speed 4.5 m/s. The instability at speed 4.5 m/s is stronger than that at hovering flight.

In Taylor and Thomas’ study, aerodynamic derivatives were measured from a “flying” locust^1^. In this type of experiment, control responses of the insect must have been introduced to the measurement and inherent (or passive) stability derivatives cannot be obtained due to the tethering. Thus, the results they obtained may not represent the passive stability of the insect. In the bumblebee study of Xiong and Sun^13^ and Xu and Sun^14^, passive stability derivatives were obtained by computation; but the flight-speed range was limited to 0–4.5 m/s, because flight data were only measured in this speed range^15^. As described above, at velocity of 4.5 m/s, the motion is more unstable. What the flight stability properties would become at higher speeds is unknown. Stability properties for the full range of speed of an insect are of great interest.

Recently, our group measured the flapping kinematics of freely-flying droneflies^16^. The speed range is 0–8.6 m/s. It was shown that the insect could not fly at speeds higher than about 8.6 m/s and therefore the above flight speed range could represent the full speed range of the insect. The reason for choosing droneflies for the experiment was that they fly very well in the experiment conditions. And moreover, the size of dronefly is approximately the same as that of the medium-sized bumblebee and results can therefore be compared between the two. Here, we investigate the stability properties of a forward-flying dronefly in the full speed range of the insect (0–8.6 m/s), using the above mentioned measured data, including hover flight. With this study, we can answer the above question that at higher flight speed, the flight will become more unstable or less unstable.

## Materials and Methods

As in our previous works in this area^2,13,14^, averaged-model (see below) is used to represent the dynamics of the insect and linearization theory is applied to the problem. We solve the Navier-Stokes equations numerically to obtain the aerodynamic derivatives, so that inherent (or passive) stability derivative can be obtained. At each flight speed, first, aerodynamic forces and moments at balanced flight are calculated using the measured flapping kinematics from Meng and Sun^16^. Then we compute the aerodynamic derivatives. Finally, we analize the stability properties at each flight speed by examining the natural modes of motion.

### Reference frames and equations of motion

**Figure 1:**
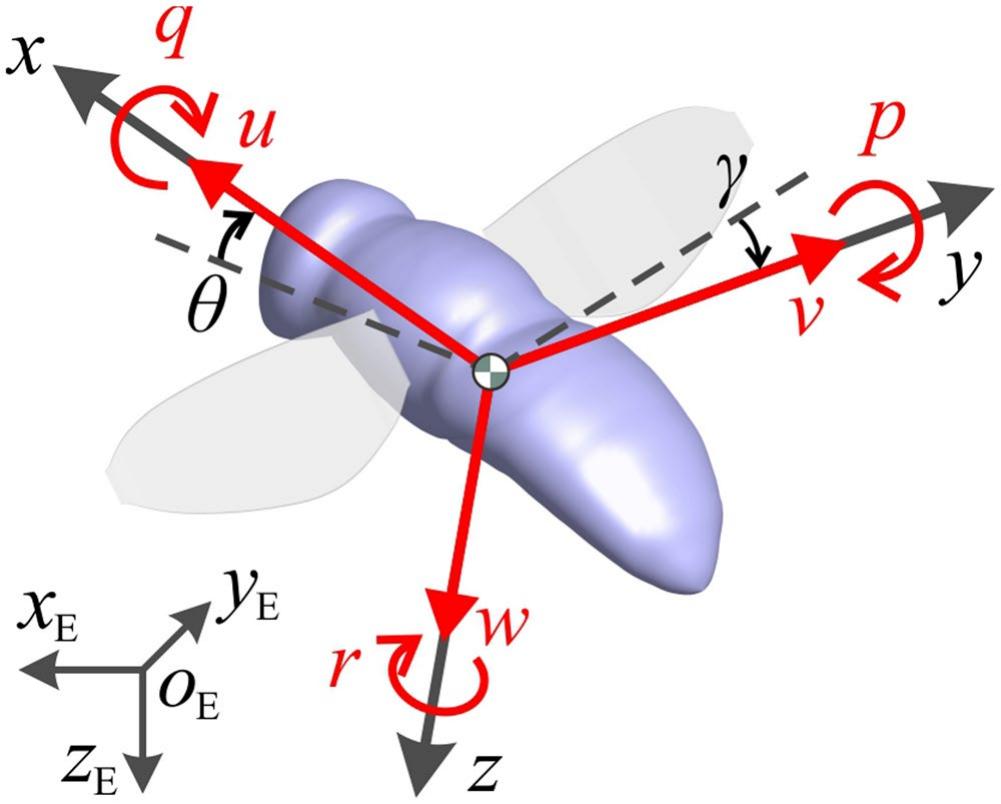
A sketch showing the reference frames and state variables. *xyz* is a non-inertial frame fixed on the body and *x*_E_*y*_E_*z*_E_ is a laboratory frame. The origin of the *xyz* frame is at the center of mass of the insect.

A flying insect is a nonlinear time-variant dynamic system. For such a system, the oscillating mass distribution and the periodic aerodynamic and inertial forces associated with the oscillating wings can couple with the body’s natural modes of motion. But if the wingbeat frequency is sufficiently high compared to the insect’s natural frequencies of gross motion, such a coupling is unlikely to occur. In this case, one may consider replacing the forces by wingbeat-cycle average forces, which might vary over the time scale of the gross motion of the insect. This is referred to as averaged-model. Detailed description of the averaged-model can be found in Taylor and Thomas^1^ and Sun and Xiong^2^. In the averaged-model, the insect is represented by rigid body (Fig. 1); the aerodynamic forces and moments of the wing are time-averaged over each flapping period; these mean forces and moments vary with time during the disturbed motion.

Thus the equations of motion is similar to those of an airplane or helicopter, which can be found in Etkin and Reid’s book on flight dynamics of airplanes^17^ (or any other textbook on this subject):

1 $$X-mg\,\sin \,\theta =m(\dot{u}+wq-vr)$$

2 $$Y+mg\,\cos \,\theta \,\sin \,\gamma =m(\dot{v}+ru-pw)$$

3 $$Z+mg\,\cos \,\theta \,\cos \,\gamma =m(\dot{w}-qu+pv)$$

4 $$L={I}_{x}\dot{p}-{I}_{xz}\dot{r}-{I}_{xz}pq-({I}_{y}-{I}_{z})qr$$

5 $$M={I}_{y}\dot{q}+{I}_{xz}({p}^{2}-{r}^{2})+({I}_{x}-{I}_{z})rq$$

6 $$N=-\,{I}_{xz}\dot{p}+{I}_{z}\dot{r}-({I}_{x}-{I}_{y})pq+{I}_{xz}qr$$

7 $$\dot{\gamma }=p+\,\tan \,\theta (q\,\sin \,\gamma +r\,\cos \,\gamma )$$

8 $$\dot{\theta }=q\,\cos \,\gamma -r\,\sin \,\gamma $$

9 $$\dot{\psi }=\frac{1}{\cos \,\theta }(q\,\sin \,\gamma +r\,\cos \,\gamma )$$

where *u* is the *x-*component of the velocity of the center of mass (Fig. 1), *v* and *w* are the corresponding *y* and *z* components, respectively; *θ* is the pitch angle, *γ* is the roll angle and *ψ* is yaw angle (Fig. 1) (*u*, *v*, *w*, *θ*, *γ* and *ψ* are referred to as state variables); *X* is the *x*-component of the aerodynamic force acting on the insect and *Y* and *Z* are the corresponding *y*- and *z*-components, respectively; *L* is the *x*-component of the aerodynamic moment, and *Y* and *Z* are the corresponding *y*- and *z*-components, respectively (note that they are contributed by both the wings and the body); *m* is insect mass; *g* is the gravity constant; *I*_x_, *I*_y_ and *I*_z_ are the moments of inertia about the *x*-, *y*- and *z*-axis, respectively, and *I*_xz_ is corresponding product of inertia; “∙” indicates taking time (*t*) derivative. We can see that Eq. (9) is decoupled from Eqs. (1)–(8) since the yaw angle (*ψ*) does not appear in Eqs. (1)–(8).

Although Eqs. (1)–(8) are non-linear, but they can be linearized by assuming the disturbance motion from the equilibrium flight is small^1,2^. That is, we write *u* = *u*_e_ + *δu*, *v* = *v*_e_ + *δv* etc., where the subscript “e” denotes the equilibrium-flight condition and the symbol “*δ*” indicates a small quantity; we also write *X* = *X*_e_ + *δX*, *Y* = *Y*_e_ + *δY* etc. The six force and moment disturbances, *δX*, *δY*, etc. are further expanded as Taylor’s series in terms of the disturbance state variables (the nonlinear terms are dropped)^1,2^:

10 $$\delta X={X}_{{\rm{u}}}\delta u+{X}_{{\rm{v}}}\delta v+{X}_{{\rm{w}}}\delta w+{X}_{{\rm{p}}}\delta p+{X}_{{\rm{q}}}\delta q+{X}_{{\rm{r}}}\delta r$$

11 $$\delta Y={Y}_{{\rm{u}}}\delta u+{Y}_{{\rm{v}}}\delta v+{Y}_{{\rm{w}}}\delta w+{Y}_{{\rm{p}}}\delta p+{Y}_{{\rm{q}}}\delta q+{Y}_{{\rm{r}}}\delta r$$

12 $$\delta Z={Z}_{{\rm{u}}}\delta u+{Z}_{{\rm{v}}}\delta v+{Z}_{{\rm{w}}}\delta w+{Z}_{{\rm{p}}}\delta p+{Z}_{{\rm{q}}}\delta q+{Z}_{{\rm{r}}}\delta r$$

13 $$\delta L={L}_{{\rm{u}}}\delta u+{L}_{{\rm{v}}}\delta v+{L}_{{\rm{w}}}\delta w+{L}_{{\rm{p}}}\delta p+{L}_{{\rm{q}}}\delta q+{L}_{{\rm{r}}}\delta r$$

14 $$\delta M={M}_{{\rm{u}}}\delta u+{M}_{{\rm{v}}}\delta v+{M}_{{\rm{w}}}\delta w+{M}_{{\rm{p}}}\delta p+{M}_{{\rm{q}}}\delta q+{M}_{{\rm{r}}}\delta r$$

15 $$\delta N={N}_{{\rm{u}}}\delta u+{N}_{{\rm{v}}}\delta v+{N}_{{\rm{w}}}\delta w+{N}_{{\rm{p}}}\delta p+{N}_{{\rm{q}}}\delta q+{N}_{{\rm{r}}}\delta r$$

where *X*_u_, *X*_v_, etc. are the aerodynamic or stability derivatives. Since the insect is symmetrical with respect to the vertical plane passing the longitudinal axis, the longitudinal disturbance motion (*δu*, *δw*, *δq* and *δθ*) will not produce lateral aerodynamic forces and moments, thus the lateral aerodynamic derivatives due to longitudinal disturbance motion are zero, i.e., *Y*_u_ = *Y*_w_ = *Y*_q_ = *L*_u_ = *L*_w_ = *L*_q_ = *N*_u_ = *N*_w_ = *N*_q_ = 0. Therefore, Eqs. (10)–(15) can be simplified as:

16 $$\delta X={X}_{{\rm{u}}}\delta u+{X}_{{\rm{v}}}\delta v+{X}_{{\rm{w}}}\delta w+{X}_{{\rm{p}}}\delta p+{X}_{{\rm{q}}}\delta q+{X}_{{\rm{r}}}\delta r$$

17 $$\delta Y={Y}_{{\rm{v}}}\delta v+{Y}_{{\rm{p}}}\delta p+{Y}_{{\rm{r}}}\delta r$$

18 $$\delta Z={Z}_{{\rm{u}}}\delta u+{Z}_{{\rm{v}}}\delta v+{Z}_{{\rm{w}}}\delta w+{Z}_{{\rm{p}}}\delta p+{Z}_{{\rm{q}}}\delta q+{Z}_{{\rm{r}}}\delta r$$

19 $$\delta L={L}_{{\rm{v}}}\delta v+{L}_{{\rm{p}}}\delta p+{L}_{{\rm{r}}}\delta r$$

20 $$\delta M={M}_{{\rm{u}}}\delta u+{M}_{{\rm{v}}}\delta v+{M}_{{\rm{w}}}\delta w+{M}_{{\rm{p}}}\delta p+{M}_{{\rm{q}}}\delta q+{M}_{{\rm{r}}}\delta r$$

21 $$\delta N={N}_{{\rm{v}}}\delta v+{N}_{{\rm{p}}}\delta p+{N}_{{\rm{r}}}\delta r$$

For a conventional airplane, it has been shown that the lateral disturbance motion (*δv*, *δp*, *δr* and *δγ*) will only produce negligibly small longitudinal disturbance^17^. Therefore, the longitudinal aerodynamic derivatives due to lateral disturbance motion are approximately zero, i.e.

22 $${X}_{{\rm{v}}}\approx 0,\,{Z}_{{\rm{v}}}\approx 0,\,{M}_{{\rm{v}}}\approx 0,\,{X}_{{\rm{p}}}\approx 0,\,{Z}_{{\rm{p}}}\approx 0,\,{M}_{{\rm{p}}}\approx 0,\,{X}_{{\rm{r}}}\approx 0,\,{Z}_{{\rm{r}}}\approx 0,\,{M}_{{\rm{r}}}\approx 0$$

and Eqs. (16)–(21) can be further simplified. For an insect in flapping flight, especially in forward flight, whether or not this is true is unknown. We will examine this in our calculation below.

At reference (or equilibrium) flight, *u*_e_* = V*_e_ (*V*_e_ is the forward flight speed) and *v*_e_, *w*_e_, *p*_e_, *q*_e_, *r*_e_, *θ*_e_, *γ*_e_ are zero (the zero *θ*_e_ and *γ*_e_ are due to the *x*-y plane being horizontal at equilibrium flight); *Z*_e_ = −*mg* (vertcal forces balance te weight); *X*_e_, *Y*_e_, *L*_e_, *M*_e_, *N*_e_ are zero (the insect is in equilibrium state). Substituting the expressions of independent state variables and Eqs. (16)–(21) into Eqs. (1)–(8), the linearized equations of motion can be obtained and they are expressed in the following non-dimensional matrix form:

23 $$[\begin{array}{c}\delta {\dot{u}}^{+}\\ \delta {\dot{w}}^{+}\\ \delta {\dot{q}}^{+}\\ \delta {\dot{\theta }}^{+}\\ \delta {\dot{v}}^{+}\\ \delta {\dot{p}}^{+}\\ \delta {\dot{r}}^{+}\\ \delta {\dot{\gamma }}^{+}\end{array}]={\bf{A}}[\begin{array}{c}\delta {u}^{+}\\ \delta {w}^{+}\\ \delta {q}^{+}\\ \delta {\theta }^{+}\\ \delta {v}^{+}\\ \delta {p}^{+}\\ \delta {r}^{+}\\ \delta {\gamma }^{+}\end{array}]$$

where **A** is referred to as system matrix:

24 $${\bf{A}}=[\begin{array}{cccccccc}\frac{{X}_{{\rm{u}}}^{+}}{{m}^{+}} & \frac{{X}_{{\rm{w}}}^{+}}{{m}^{+}} & \frac{{X}_{{\rm{q}}}^{+}}{{m}^{+}} & -{g}^{+} & \frac{{X}_{{\rm{v}}}^{+}}{{m}^{+}} & \frac{{X}_{{\rm{p}}}^{+}}{{m}^{+}} & \frac{{X}_{{\rm{r}}}^{+}}{{m}^{+}} & 0\\ \frac{{Z}_{{\rm{u}}}^{+}}{{m}^{+}} & \frac{{Z}_{{\rm{w}}}^{+}}{{m}^{+}} & \frac{{Z}_{{\rm{q}}}^{+}}{{m}^{+}}+{V}_{{\rm{e}}}^{+} & 0 & \frac{{Z}_{{\rm{v}}}^{+}}{{m}^{+}} & \frac{{Z}_{{\rm{p}}}^{+}}{{m}^{+}} & \frac{{Z}_{{\rm{r}}}^{+}}{{m}^{+}} & 0\\ \frac{{M}_{{\rm{u}}}^{+}}{{I}_{{\rm{y}}}^{+}} & \frac{{M}_{{\rm{w}}}^{+}}{{I}_{{\rm{y}}}^{+}} & \frac{{M}_{{\rm{q}}}^{+}}{{I}_{{\rm{y}}}^{+}} & 0 & \frac{{M}_{{\rm{v}}}^{+}}{{I}_{{\rm{y}}}^{+}} & \frac{{M}_{{\rm{p}}}^{+}}{{I}_{{\rm{y}}}^{+}} & \frac{{M}_{{\rm{r}}}^{+}}{{I}_{{\rm{y}}}^{+}} & 0\\ 0 & 0 & 1 & 0 & 0 & 0 & 0 & 0\\ 0 & 0 & 0 & 0 & \frac{{Y}_{{\rm{v}}}^{+}}{{m}^{+}} & \frac{{Y}_{{\rm{p}}}^{+}}{{m}^{+}} & \frac{{Y}_{{\rm{r}}}^{+}}{{m}^{+}}-{V}_{{\rm{e}}}^{+} & {g}^{+}\\ 0 & 0 & 0 & 0 & \frac{{I}_{{\rm{z}}}^{+}{L}_{{\rm{v}}}^{+}+{I}_{{\rm{xz}}}^{+}{N}_{{\rm{v}}}^{+}}{{I}_{{\rm{x}}}^{+}{I}_{{\rm{z}}}^{+}-{I}_{{\rm{xz}}}^{+\,2}} & \frac{{I}_{{\rm{z}}}^{+}{L}_{{\rm{p}}}^{+}+{I}_{{\rm{xz}}}^{+}{N}_{{\rm{p}}}^{+}}{{I}_{{\rm{x}}}^{+}{I}_{{\rm{z}}}^{+}-{I}_{{\rm{xz}}}^{+\,2}} & \frac{{I}_{{\rm{z}}}^{+}{L}_{{\rm{r}}}^{+}+{I}_{{\rm{xz}}}^{+}{N}_{{\rm{r}}}^{+}}{{I}_{{\rm{x}}}^{+}{I}_{{\rm{z}}}^{+}-{I}_{{\rm{xz}}}^{+\,2}} & 0\\ 0 & 0 & 0 & 0 & \frac{{I}_{{\rm{xz}}}^{+}{L}_{{\rm{v}}}^{+}+{I}_{{\rm{x}}}^{+}{N}_{{\rm{v}}}^{+}}{{I}_{{\rm{x}}}^{+}{I}_{{\rm{z}}}^{+}-{I}_{{\rm{xz}}}^{+\,2}} & \frac{{I}_{{\rm{xz}}}^{+}{L}_{{\rm{p}}}^{+}+{I}_{{\rm{x}}}^{+}{N}_{{\rm{p}}}^{+}}{{I}_{{\rm{x}}}^{+}{I}_{{\rm{z}}}^{+}-{I}_{{\rm{xz}}}^{+\,2}} & \frac{{I}_{{\rm{xz}}}^{+}{L}_{{\rm{r}}}^{+}+{I}_{{\rm{x}}}^{+}{N}_{{\rm{r}}}^{+}}{{I}_{{\rm{x}}}^{+}{I}_{{\rm{z}}}^{+}-{I}_{{\rm{xz}}}^{+\,2}} & 0\\ 0 & 0 & 0 & 0 & 0 & 1 & 0 & 0\end{array}]$$

When non-dimensionalizing the equations, we take the mean wing-chord length (*c*) as the reference length, the mean flapping velocity at hovering flight (*U*) as the reference velocity (here *U* = 2*Φnr*_2_*Φ* is the stroke amplitude at hovering flight, *r*_2_ is the radius of the second moment of wing area and *n* is the flapping frequency at hovering), and *T* = 1/*n* as the reference time. The non-dimensional forms of the variables are: *V*_e_^+^ = *V*_e_/*U*, *δu*^+^ = *δu*/*U, δv*^+^ = *δv*/*U, δw*^+^ = *δw*/*U, δp*^+^ = *δpT, δq*^+^ = *δqT, δr*^+^ = *δrT, X*^+^ = *X*/0.5*ρU*^2^*S*_t_ (here *ρ* is the air density; *S*_t_ is the area of the wing-pair), *Y*^+^ = *Y*/0.5*ρU*^2^*S*_t_, *Z*^+^ = *Z*/0.5*ρU*^2^*S*_t_, *L*^+^ = *L*/0.5*ρU*^2^*S*_t_*c*, *M*^+^ = *M*/0.5*ρU*^2^*S*_t_*c*, *N*^+^ = *N*/0.5*ρU*^2^*S*_t_*c*, *t*^+^ = *t*/*T*, *m*^+^ = *m*/0.5*ρUS*_t_*T*, *I*_x_^+^ = *I*_x_/0.5*ρU*^2^*S*_t_*cT*^2^, *I*_y_^+^ = *I*_y_/0.5*ρU*^2^*S*_t_*cT*^2^, *I*_z_^+^ = *I*_z_/0.5*ρU*^2^*S*_t_*cT*^2^, *I*_xz_^+^ = *I*_xz_/0.5*ρU*^2^*S*_t_*cT*^2^ and *g*^+^ = *gT*/*U*.

### Flight data

**Figure 2:**
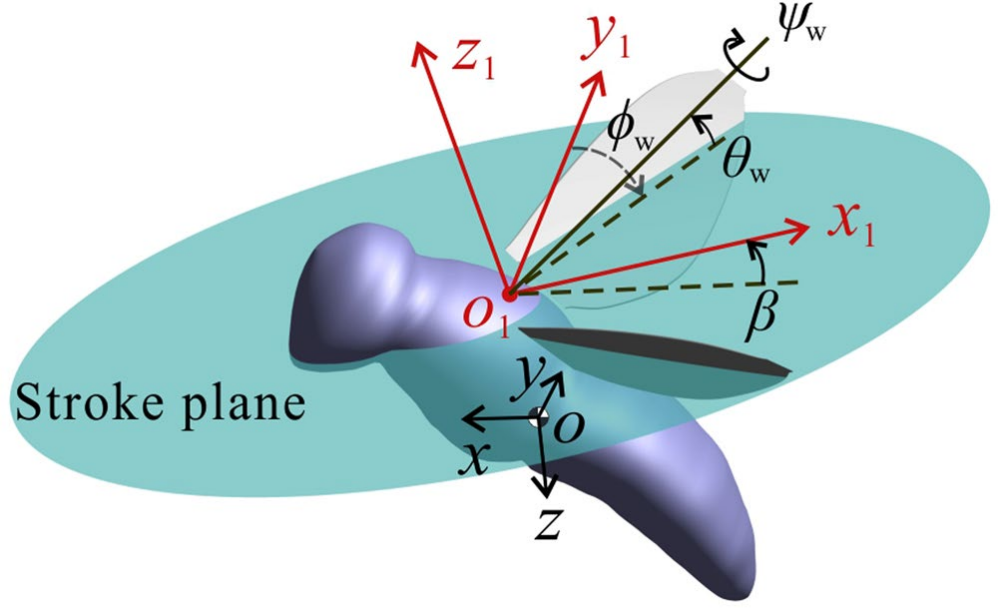
Definitions of the wing kinematics.

To solve Eqs. (23) and (24), we need aerodynamic derivatives, insect mass and insect moments of inertia and product of inertia. The mass and the moments and product of inertia are available from ref. ^16^ or can be computed using data given in ref. ^16^. The aerodynamic or stability derivatives are computed in the present study. Wing kinematics are needed for computing the aerodynamic derivatives. Wing kinematics of the dronefly is available in ref. ^16^. For the wing-kinematics description, we follow Meng and Sun^16^: The stroke plane and three flapping angles are defined as shown in Fig. 2. *o*_1_*x*_1_*y*_1_*z*_1_ is a coordinate system with *o*_1_ at the wing-root and *x*_1_ and *y*_1_ in the stroke plane. The stroke plane angle is denoted as *β* and it is the angle between the horizontal plane and the stroke plane when the insect is at equilibrium flight. The flapping angles include *ϕ*_w_, *θ*_w_ and *ψ*_w_: *ϕ*_w_ is the positional angle, *θ*_w_ is the stroke deviation angle, *ψ*_w_ is the pitch angle. The detailed description of *ϕ*_w_, *θ*_w_ and *ψ*_w_ can be found in Taylor and Thomas^1^ and Sun and Xiong^2^. With *β*, *ϕ*_w_, *θ*_w_ and *ψ*_w_ known, the wing-kinematics is determined. Body orientation is determined by the body angle *χ*, which is the angle between body and the horizontal.

**Figure 3:**
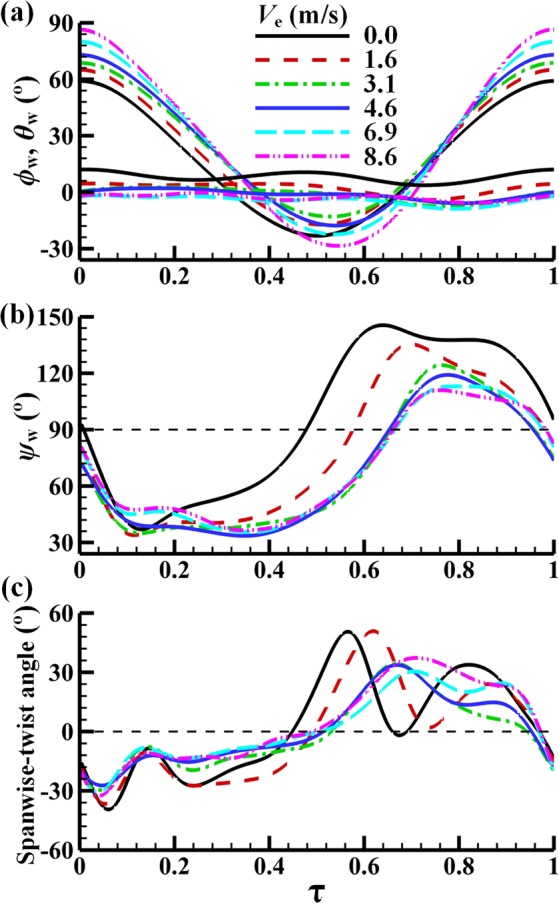
Wing motion of dronefly in a flapping period; data for six flight speeds. *τ* = 0 is the start of a downstroke; *τ* = 1 is the end of the following upstroke.

Values of the measured *ϕ*_*w*_, *θ*_*w*_, *ψ*_*w*_ and spanwise-twist angle in one flapping cycle for the dronefly are shown in Fig. 3. The flapping amplitude, *Φ*, is defined as the difference between the maximum *ϕ*_w_ and the minimum *ϕ*_w_. The values of *Φ*, *n*, *β* and *χ* are listed in Table 1; they are needed in the non-dimensionalization and in the computations.

**Table 1:** Kinematic parameters of the dronefly.

*V*_e_ (m/s)	*n* (Hz)	*Φ* (°)	*β* (°)	*χ* (°)
0.0	178	85	11	30
1.6	166	83	19	24
3.1	164	82	26	17
4.6	167	92	37	7
6.9	173	103	46	3
8.6	180	115	50	1

The morphological parameters are: *m* = 151 mg; wing-length *R = *13.05 mm; *c = *3.31 mm, *r*_2_/*R = *0.55; single wing area *S = *43.23 mm^2^; body length *l*_b_ = 16.21 mm; distance from head to center of mass divided by body length is *l*_2_/*l*_b_ = 0.48; distance from wing base axis to center of mass divided by body length *l*_1_/*l*_b_ = 0.12; distance between the two wing roots divided by body length *l*_r_/*l*_b_ = 0.29; the free body angle *χ*_0_ = 59.5°. *I*_x_, *I*_y_, *I*_z_ and *I*_xz_ are calculated using the flight data given in Meng and Sun^16^, using the method described in our previous papers^14,18^. Note that at different flight speed the body position relative to the *oxyz* coordinate frame is different, therefore, *I*_x_, *I*_y_, *I*_z_ and *I*_xz_ are different for different flight speed; the values are given in Table 2.

**Table 2:** The moments of inertia and the products of inertia.

*V*_e_ (m/s)	*I*_x_ (mg·mm^2^)	*I*_y_ (mg·mm^2^)	*I*_z_ (mg·mm^2^)	*I*_xz_ (mg·mm^2^)
0.0	987.1	2699.7	2379.1	971.7
1.6	796.6	2698.9	2559.5	804.4
3.1	625.2	2698.9	2723.5	566.7
4.6	494.9	2698.9	2853.7	173.7
6.9	482.8	2699.0	2863.9	7.9
8.6	485.7	2699.0	2861.0	−75.0

### Calculation of the aerodynamic derivatives

The aerodynamic derivatives are calculated by solution of the flow equations (the Navier-Stokes equations). Moving overset grids are employed owing to the relative motion of the wings and body. The flow solver is the same as that employed in several previous works of our group^14,19,20^ and the description of the method is presented in the Supplementary Material, Supplementary S1. Tests of the computational grid are also given in the Supplementary Material, Supplementary S2.

### Solving the equations of motion

After the morphological parameters and flight data are given and the aerodynamic derivatives are calculated, the matrixes **A** in Eq. (23) are determined. We are ready to solve Eq. (23) and analyze the flight stability of the dronefly.

Let ***x*** be the state variable vector in Eq. (23):

25 $${\boldsymbol{x}}=[\begin{array}{c}\delta u\\ \delta w\\ \delta q\\ \delta \theta \\ \delta v\\ \delta p\\ \delta r\\ \delta \gamma \end{array}]$$

The solution of Eq. (23) is^17^:

26 $${\boldsymbol{x}}=\mathop{\sum }\limits_{i=1}^{8}{{\boldsymbol{a}}}_{{\rm{i}}}{e}^{{\lambda }_{{\rm{i}}}{t}^{+}}$$

where *λ*_i_ denotes an eigenvalue of **A** and ***a***_i_ is a vector determined by the corresponding eigenvector. From Eq. (26), it is clear that whether or not the solution or the flight is stable is decided by the eigenvalues *λ*_i_ (*i* = 1–8). *λ*_i_ can be real or complex numbers (complex eigenvalues occur as conjugate pairs). Each real eigenvalue or each pair of complex eigenvalues gives a simple motion, and the solution is a linear combination of the simple motions. A simple motion is referred to as a natural mode of motion. If the one or several real eigenvalues are positive, or the real part of one or several complex eigenvalues are positive, the flight will be unstable.

## Results and Discussion

### The equilibrium flight

**Figure 4:**
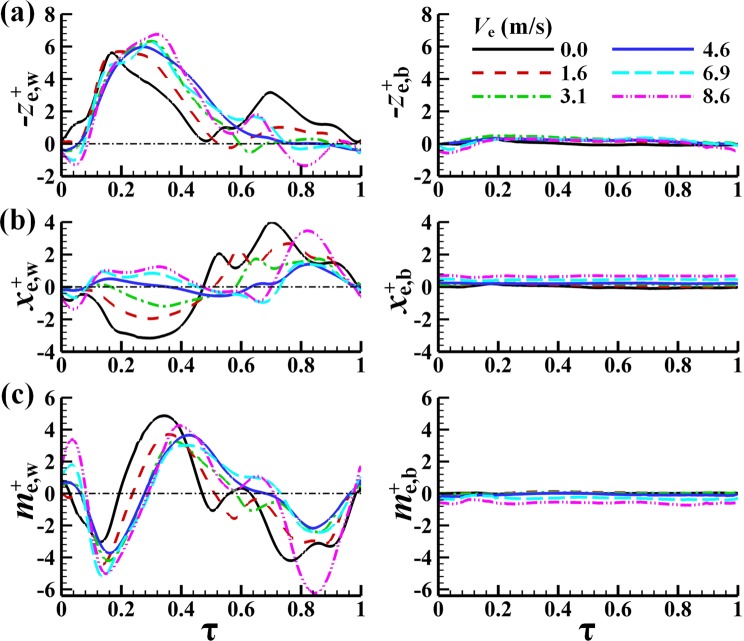
Time variations of the aerodynamic forces and moment of the wings and the body as functions of time in a flapping period (equilibrium flight).

With the wing kinematics data at equilibrium flight given above, the aerodynamic forces at each flight speed can be computed. These are the aerodynamic forces at equilibrium flight. We use *x*_e_^+^, *z*_e_^+^ and *m*_e_^+^ to represent the instantaneous non-dimensional forces and pitch moment at equilibrium flight. *x*_e_^+^ is the horizontal force. *z*_e_^+^ is the vertical force. *m*_e_^+^ is the pitch moment. At equilibrium flight, because of the symmetry of the flow, only *x*_e_^+^, *z*_e_^+^ and *m*_e_^+^ are non-zero. The non-dimensional wingbeat-cycle averages of *x*_e_^+^, *z*_e_^+^ and *m*_e_^+^ are *X*_e_^+^, *Z*_e_^+^ and *M*_e_^+^, respectively. Subscript “w” denotes the contributions of the wing. Subscript “b” denotes the contributions of the body. Figure 4 shows the computed ${x}_{e,w}^{+}$, ${x}_{e,b}^{+}$, ${z}_{e,w}^{+}$, ${z}_{e,b}^{+}$, ${m}_{e,w}^{+}$ and ${m}_{e,b}^{+}$ in a flapping cycle at various flight speeds, after periodical state has been established.

The following observations can be made from Fig. 4. At hovering (*V*_e_ = 0 m/s, Fig. 4), positive vertical force (negative ${z}_{e,w}^{+}$) is produced in down- and upstrokes and $-{z}_{e,w}^{+}$ in the downstroke is slightly larger than its counterpart in the upstroke. Both the down- and upstrokes contribute to the cycle-average force that supports the weight. Positive ${x}_{e,w}^{+}$ is produced in the upstroke and negative ${x}_{e,w}^{+}$ in the downstroke, and they have almost the same magnitude, resulting in an approximately zero mean horizontal force, as required for the hovering flight. At low speed (*V*_e_ = 1.6 m/s, Fig. 4), $-{z}_{e,w}^{+}$ in the downstroke is much larger than its counterpart in the upstroke. Moreover, the downstroke duration is longer than that of the hovering case. Thus, the weight-supporting force is mainly generated by the downstroke. Positive ${x}_{e,w}^{+}$ is still generated in the upstroke and negative ${x}_{e,w}^{+}$ in the downstroke, but the magnitude of the negative ${x}_{e,w}^{+}$ in the downstroke is smaller than that of the positive ${x}_{e,w}^{+}$ in the upstroke, giving a positive cycle-average thrust. This shows that the force required for overcoming the drag of insect body is generated by the upstroke. At medium speed (*V*_e_ = 3.1 m/s, Fig. 4), $-{z}_{e,w}^{+}$ is rather large during the downstroke but close to zero during the upstroke. Now the weight-supporting force is produced during the downstroke. As in the low-speed case, the force required for overcoming the drag of insect body is generated by the upstroke. At fast forward flight speeds (*V*_e_ = 4.6–8.6 m/s, Fig. 4), as in the medium-speed case, $-{z}_{e,w}^{+}$ is large in the downstroke and close to zero (even negative at 8.6 m/s) in the upstroke, and the downstroke duration is even longer. Again, the weight-supporting force is produced in the downstroke. Unlike the medium-speed case, a large part of the downstroke (*τ = *0.09–0.47, Fig. 4) also produces positive ${x}_{e,w}^{+}$, showing that the thrust required for overcoming the body drag is produced by both the down- and upstrokes.

**Figure 5:**
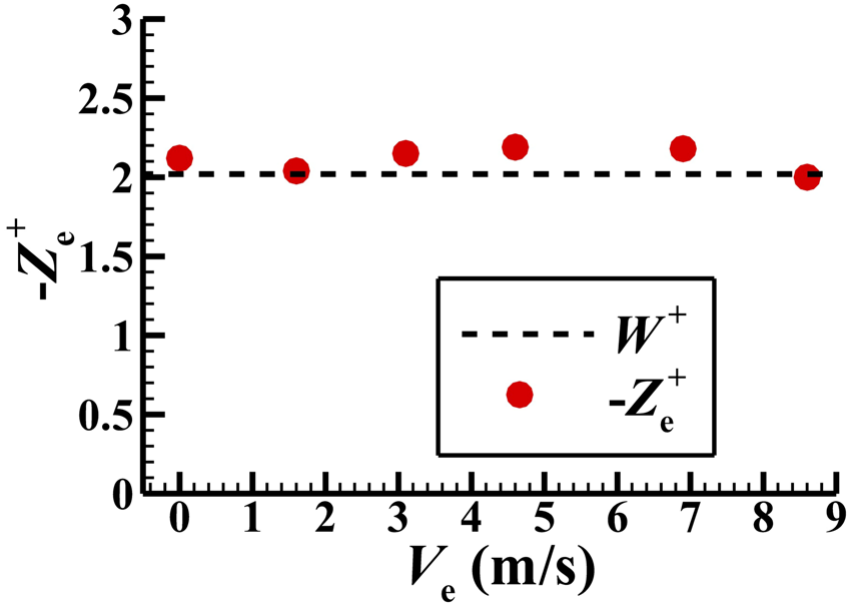
The calculated mean non-dimensional vertical force at various flight speeds, compared with the non-dimensional weight.

For constant-speed flight, the aerodynamic force in the vertical direction should approximately equal the insect-weight. The weight of the flies has been measured^16^ and the non-dimensionalized weight (*W*^+^) is 2.02. The computed non-dimensional vertical force of the wings plus that of the body, $-{Z}_{{\rm{e}}}^{+}$, for various flight speeds are shown in Fig. 5, compared with the non-dimensional weight *W*^+^. At four flight speeds, the computed value is different from the measured by no more than 6%; at *V*_e_ = 4.6 m/s and 6.9 m/s, the differences are about 8%. We see that the requirement that the vertical force balances the weight is approximately satisfied.

### The aerodynamic derivatives

**Figure 6:**
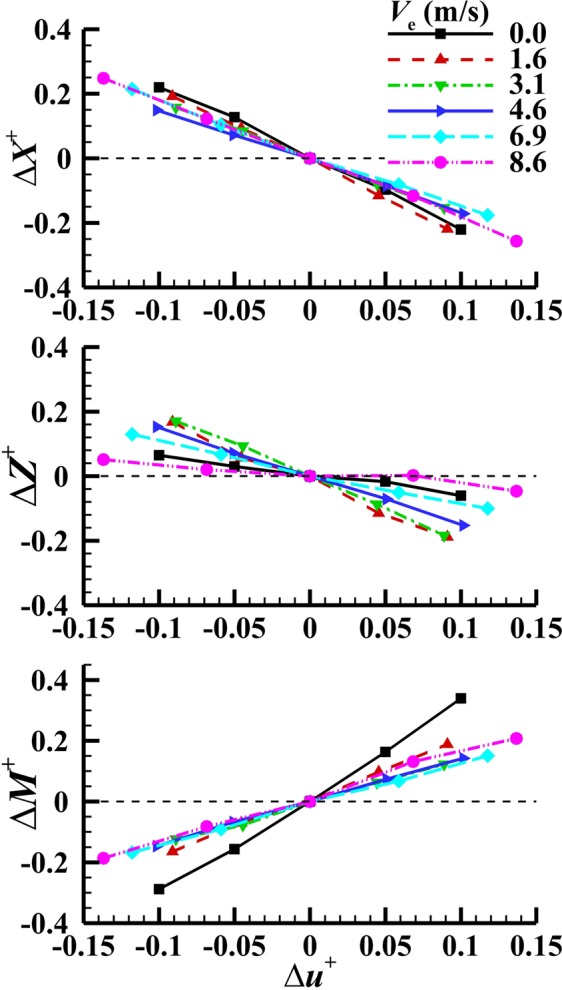
The *u*-series force and moment data.

To compute the aerodynamic derivatives with respect to a state variable, e.g. *u*, we vary the state variable *u* from its equilibrium value (other state variables are kept unchanged) and calculate the aerodynamic forces, and moments, *X*^+^, *Y*^+^, *Z*^+^ *L*^+^, *M*^+^ and *N*^+^. As an example, the computed *X*^+^, *Z*^+^ and *M*^+^ are shown in Fig. 6 (“Δ” in the figure represents the difference between a variable and its equilibrium value). It is seen that *X*^+^, *Z*^+^ and *M*^+^ vary with *u*^+^ approximately linearly when −0.15 ≤ Δ*u*^+^ ≤ 0.15 (Fig. 6). This true for the cases of varying other state variables, justifying the linear theory for small perturbation motion. From the above results, aerodynamic derivatives can be computed. The computed aerodynamic derivatives, *X*_u_^+^, *Z*_u_^+^, *M*_u_^+^, *X*_w_^+^, *Z*_w_^+^, *M*_w_^+^, *X*_q_^+^, *Z*_q_^+^, *M*_q_^+^, *X*_v_^+^, *Y*_v_^+^, *Z*_v_^+^, *L*_v_^+^, *M*_v_^+^, *N*_v_^+^, *X*_p_^+^, *Y*_p_^+^, *Z*_p_^+^, *L*_p_^+^, *M*_p_^+^, *N*_p_^+^, *X*_r_^+^, *Y*_r_^+^, *Z*_r_^+^, *L*_r_^+^, *M*_r_^+^ and *N*_r_^+^ are list in Table 3.

**Table 3:** Aerodynamic derivatives.

Longitudinal derivatives									
*V*_e_ (m/s)	*X*_u_^+^	*Z*_u_^+^	*M*_u_^+^	*X*_w_^+^	*Z*_w_^+^	*M*_w_^+^	*X*_q_^+^	*Z*_q_^+^	*M*_q_^+^
0.0	−2.221	−0.550	3.171	0.269	−1.727	−0.573	−0.225	−0.027	−0.322
1.6	−2.272	−1.917	1.892	0.107	−2.638	−0.277	−0.131	0.041	−0.368
3.1	−1.789	−2.011	1.482	−0.086	−3.721	−0.306	−0.035	−0.010	−0.499
4.6	−1.564	−1.453	1.408	0.240	−4.585	0.611	−0.065	−0.043	−0.571
6.9	−1.616	−0.989	1.349	0.192	−6.595	1.572	0.035	−0.133	−1.019
8.6	−1.794	−0.245	1.638	0.431	−7.224	2.698	0.092	−0.228	−1.305
**Lateral derivatives**									
***V***_**e**_ **(m/s)**	***Y***_**v**_^**+**^	***L***_**v**_^**+**^	***N***_**v**_^**+**^	***Y***_**p**_^**+**^	***L***_**p**_^**+**^	***N***_**p**_^**+**^	***Y***_**r**_^**+**^	***L***_**r**_^**+**^	***N***_**r**_^**+**^
0.0	−0.932	−0.520	1.007	−0.460	−2.486	−0.413	0.255	0.843	−4.338
1.6	−1.145	−1.070	2.081	−0.278	−2.765	−0.009	0.432	1.816	−2.913
3.1	−1.881	−2.585	2.177	−0.480	−3.165	0.375	0.458	2.188	−2.046
4.6	−1.998	−2.486	1.140	−0.694	−4.653	0.340	0.388	2.770	−2.005
6.9	−2.950	−4.544	1.544	−0.917	−7.364	0.832	0.326	2.892	−2.057
8.6	−3.981	−6.006	1.342	−1.086	−9.675	0.904	0.552	2.937	−2.491
**Longitudinal derivatives due to lateral motion**									
***V***_**e**_ **(m/s)**	***X***_**v**_^**+**^	***Z***_**v**_^**+**^	***M***_**v**_^**+**^	***X***_**p**_^**+**^	***Z***_**p**_^**+**^	***M***_**p**_^**+**^	***X***_**r**_^**+**^	***Z***_**r**_^**+**^	***M***_**r**_^**+**^
0.0	0.004	−0.012	−0.005	−0.001	−0.003	0.003	−0.003	−0.002	−0.001
1.6	0.003	−0.005	0.004	0.001	0.005	−0.002	−0.003	−0.002	0.002
3.1	−0.004	−0.020	0.001	−0.002	0.002	0.001	−0.002	−0.006	−0.002
4.6	−0.003	−0.018	0.003	−0.002	−0.003	0.002	0.004	0.008	0.005
6.9	−0.002	−0.001	0.001	0.000	0.006	0.003	0.008	0.004	0.000
8.6	0.000	−0.007	−0.012	0.002	0.002	0.007	0.013	0.002	−0.006

It is seen that the longitudinal derivatives due to the lateral motion are approximately 3 orders of magnitude smaller than other longitudinal derivatives (Table 3). As a result, the elements in the right and upper part of the system matrix **A** in Eq. (24) are approximately zero and **A** can be written as

27 $${\bf{A}}\approx [\begin{array}{cccccccc}\frac{{X}_{{\rm{u}}}^{+}}{{m}^{+}} & \frac{{X}_{{\rm{w}}}^{+}}{{m}^{+}} & \frac{{X}_{{\rm{q}}}^{+}}{{m}^{+}} & -{g}^{+} & 0 & 0 & 0 & 0\\ \frac{{Z}_{{\rm{u}}}^{+}}{{m}^{+}} & \frac{{Z}_{{\rm{w}}}^{+}}{{m}^{+}} & \frac{{Z}_{{\rm{q}}}^{+}}{{m}^{+}}+{V}_{{\rm{e}}}^{+} & 0 & 0 & 0 & 0 & 0\\ \frac{{M}_{{\rm{u}}}^{+}}{{I}_{{\rm{y}}}^{+}} & \frac{{M}_{{\rm{w}}}^{+}}{{I}_{{\rm{y}}}^{+}} & \frac{{M}_{{\rm{q}}}^{+}}{{I}_{{\rm{y}}}^{+}} & 0 & 0 & 0 & 0 & 0\\ 0 & 0 & 1 & 0 & 0 & 0 & 0 & 0\\ 0 & 0 & 0 & 0 & \frac{{Y}_{{\rm{v}}}^{+}}{{m}^{+}} & \frac{{Y}_{{\rm{p}}}^{+}}{{m}^{+}} & \frac{{Y}_{{\rm{r}}}^{+}}{{m}^{+}}-{V}_{{\rm{e}}}^{+} & {g}^{+}\\ 0 & 0 & 0 & 0 & \frac{{I}_{{\rm{z}}}^{+}{L}_{{\rm{v}}}^{+}+{I}_{{\rm{xz}}}^{+}{N}_{{\rm{v}}}^{+}}{{I}_{{\rm{x}}}^{+}{I}_{{\rm{z}}}^{+}-{I}_{{\rm{xz}}}^{+\,2}} & \frac{{I}_{{\rm{z}}}^{+}{L}_{{\rm{p}}}^{+}+{I}_{{\rm{xz}}}^{+}{N}_{{\rm{p}}}^{+}}{{I}_{{\rm{x}}}^{+}{I}_{{\rm{z}}}^{+}-{I}_{{\rm{xz}}}^{+\,2}} & \frac{{I}_{{\rm{z}}}^{+}{L}_{{\rm{r}}}^{+}+{I}_{{\rm{xz}}}^{+}{N}_{{\rm{r}}}^{+}}{{I}_{{\rm{x}}}^{+}{I}_{{\rm{z}}}^{+}-{I}_{{\rm{xz}}}^{+\,2}} & 0\\ 0 & 0 & 0 & 0 & \frac{{I}_{{\rm{xz}}}^{+}{L}_{{\rm{v}}}^{+}+{I}_{{\rm{x}}}^{+}{N}_{{\rm{v}}}^{+}}{{I}_{{\rm{x}}}^{+}{I}_{{\rm{z}}}^{+}-{I}_{{\rm{xz}}}^{+\,2}} & \frac{{I}_{{\rm{xz}}}^{+}{L}_{{\rm{p}}}^{+}+{I}_{{\rm{x}}}^{+}{N}_{{\rm{p}}}^{+}}{{I}_{{\rm{x}}}^{+}{I}_{{\rm{z}}}^{+}-{I}_{{\rm{xz}}}^{+\,2}} & \frac{{I}_{{\rm{xz}}}^{+}{L}_{{\rm{r}}}^{+}+{I}_{{\rm{x}}}^{+}{N}_{{\rm{r}}}^{+}}{{I}_{{\rm{x}}}^{+}{I}_{{\rm{z}}}^{+}-{I}_{{\rm{xz}}}^{+\,2}} & 0\\ 0 & 0 & 0 & 0 & 0 & 1 & 0 & 0\end{array}]$$

Thus, as in the case of an airplane or helicopter, the linearization equations of motion of the insect can be separated into two equations, one giving longitudinal disturbance motion and the other giving lateral disturbance motion. The longitudinal one is:

28 $$[\begin{array}{c}\delta {\dot{u}}^{+}\\ \delta {\dot{w}}^{+}\\ \delta {\dot{q}}^{+}\\ \delta \dot{\theta }\end{array}]={{\bf{A}}}_{1}[\begin{array}{c}\delta {u}^{+}\\ \delta {w}^{+}\\ \delta {q}^{+}\\ \delta \theta \end{array}]$$

where

29 $${{\bf{A}}}_{1}=[\begin{array}{cccc}\frac{{X}_{{\rm{u}}}^{+}}{{m}^{+}} & \frac{{X}_{{\rm{w}}}^{+}}{{m}^{+}} & \frac{{X}_{{\rm{q}}}^{+}}{{m}^{+}} & -{g}^{+}\\ \frac{{Z}_{{\rm{u}}}^{+}}{{m}^{+}} & \frac{{Z}_{{\rm{w}}}^{+}}{{m}^{+}} & \frac{{Z}_{{\rm{q}}}^{+}}{{m}^{+}}+{V}_{{\rm{e}}}^{+} & 0\\ \frac{{M}_{{\rm{u}}}^{+}}{{I}_{{\rm{y}}}^{+}} & \frac{{M}_{{\rm{w}}}^{+}}{{I}_{{\rm{y}}}^{+}} & \frac{{M}_{{\rm{q}}}^{+}}{{I}_{{\rm{y}}}^{+}} & 0\\ 0 & 0 & 1 & 0\end{array}]$$

and the lateral one is:

30 $$[\begin{array}{c}\delta {\dot{v}}^{+}\\ \delta {\dot{p}}^{+}\\ \delta {\dot{r}}^{+}\\ \delta \dot{\gamma }\end{array}]={{\bf{A}}}_{2}[\begin{array}{c}\delta {v}^{+}\\ \delta {p}^{+}\\ \delta {r}^{+}\\ \delta \gamma \end{array}]$$

where

31 $${{\bf{A}}}_{2}=[\begin{array}{cccc}\frac{{Y}_{{\rm{v}}}^{+}}{m} & \frac{{Y}_{{\rm{p}}}^{+}}{m} & \frac{{Y}_{{\rm{r}}}^{+}}{m}-{V}_{{\rm{e}}}^{+} & {g}^{+}\\ \frac{{I}_{{\rm{z}}}^{+}{L}_{{\rm{v}}}^{+}+{I}_{{\rm{xz}}}^{+}{N}_{{\rm{v}}}^{+}}{{I}_{{\rm{x}}}^{+}{I}_{{\rm{z}}}^{+}-{I}_{{\rm{xz}}}^{+\,2}} & \frac{{I}_{{\rm{z}}}^{+}{L}_{{\rm{p}}}^{+}+{I}_{{\rm{xz}}}^{+}{N}_{{\rm{p}}}^{+}}{{I}_{{\rm{x}}}^{+}{I}_{{\rm{z}}}^{+}-{I}_{{\rm{xz}}}^{+\,2}} & \frac{{I}_{{\rm{z}}}^{+}{L}_{{\rm{v}}}^{+}+{I}_{{\rm{xz}}}^{+}{N}_{{\rm{v}}}^{+}}{{I}_{{\rm{x}}}^{+}{I}_{{\rm{z}}}^{+}-{I}_{{\rm{xz}}}^{+\,2}} & 0\\ \frac{{I}_{{\rm{xz}}}^{+}{L}_{{\rm{v}}}^{+}+{I}_{{\rm{z}}}^{+}{N}_{{\rm{v}}}^{+}}{{I}_{{\rm{x}}}^{+}{I}_{{\rm{z}}}^{+}-{I}_{{\rm{xz}}}^{+\,2}} & \frac{{I}_{{\rm{xz}}}^{+}{L}_{{\rm{p}}}^{+}+{I}_{{\rm{z}}}^{+}{N}_{{\rm{p}}}^{+}}{{I}_{{\rm{x}}}^{+}{I}_{{\rm{z}}}^{+}-{I}_{{\rm{xz}}}^{+\,2}} & \frac{{I}_{{\rm{xz}}}^{+}{L}_{{\rm{r}}}^{+}+{I}_{{\rm{z}}}^{+}{N}_{{\rm{r}}}^{+}}{{I}_{{\rm{x}}}^{+}{I}_{{\rm{z}}}^{+}-{I}_{{\rm{xz}}}^{+\,2}} & 0\\ 0 & 0 & 1 & 0\end{array}]$$

**A**_1_ and **A**_2_ are the system matrixes of longitudinal motion and lateral motion, respectively.

From Table 3, one can see that several derivatives vary greatly as flight speed increasing. For example, *M*_w_^+^ is negative at hovering (−0.573), and slightly increases at *V*_e_ = 1.6 m/s and 3.1 m/s (−0.277 and −0.306); however, when flight speed reaches 4.6 m/s, *M*_w_^+^ becomes positive and then increases rapidly at high flight speeds (0.611, 1.572 and 2.698 at *V*_e_ = 4.6 m/s, 6.9 m/s and 8.6 m/s, respectively). The above result indicates that the dynamic stability properties may change greatly at high flight speeds.

### Flight stability

Now the derivatives (*X*_u_^+^, *Z*_u_^+^, *M*_u_^+^, etc.) have been computed and matrices **A**_1_ and **A**_2_ of Eqs. (28)–(31) become known (parameters, *m*^+^, *I*_x_^+^, etc., are calculated using the data in the section of Flight data). As discussed in the section of Solving the equations of motion, the eigenvalues of **A**_1_ and **A**_2_ give the stability properties of the dronefly. In the present paper, we use *λ* and *μ* to denote the eigenvalues of the longitudinal and lateral system matrices. The results are given in Table 4.

**Table 4:** Eigenvalues at various flight speeds.

Longitudinal eigenvalues				
*V*_e_ (m/s)	*λ*_1_	*λ*_2_	*λ*_3_	*λ*_4_
0.0	0.0469 + 0.0967i	0.0469–0.0967i	−0.1196	−0.0139
1.6	0.0307 + 0.0901i	0.0307–0.0901i	−0.0864	−0.0234
3.1	0.0167 + 0.0955i	0.0167–0.0955i	−0.0452 + 0.0109i	−0.0452–0.0109i
4.6	0.1007	0.0423	−0.1833	−0.0236
6.9	0.2490	0.0142	−0.3358	−0.0205
8.6	0.3803	0.0096	−0.4765	−0.0213
**Lateral eigenvalues**				
***V***_**e**_ **(m/s)**	***μ***_**1**_	***μ***_**2**_	***μ***_**3**_	***μ***_**4**_
0.0	0.0478	−0.0779 + 0.0504i	−0.0779–0.0504i	−0.5118
1.6	0.0029	−0.0754 + 0.1622i	−0.0754–0.1622i	−0.4926
3.1	−0.0012	−0.1129 + 0.2318i	−0.1129–0.2318i	−0.4533
4.6	−0.0039	−0.0494 + 0.2261i	−0.0494–0.2261i	−0.7811
6.9	−0.0044	−0.0403 + 0.2467i	−0.0403–0.2467i	−1.2339
8.6	−0.0078	−0.0301 + 0.2417i	−0.0301–0.2417i	−1.6376

First, we look at the case of hovering flight (*V*_e_ = 0 m/s). The results are similar to the previous results for dronefly^18^ and bumblebee^2^. The longitudinal modes (Table 4, *V*_e_ = 0 m/s): an unstable slow oscillatory mode (*λ*_1_ and *λ*_2_ = 0.0469 ± 0.0967*i*) and two stable subsidence modes, one fast (*λ*_3_ = −0.1196) and the other slow (*λ*_4_ = −0.0139). The lateral modes: an unstable slow divergence mode (*μ*_1_ = 0.0478), a stable oscillatory mode (*μ*_2_ and *μ*_3_ = −0.0779 ± 0.0504*i*) and a stable fast subsidence mode (*μ*_4_ = −0.5118). Owing to the two unstable modes given by *λ*_1_ and *λ*_2_ and by *μ*_1_, hovering (*V*_e_ = 0 m/s) is not stable. But the instability is weak: the disturbance doubling time (*t*_d_) is 83.0 ms for the longitudinal motion (*t*_d_ = 0.693/|*σ*|, *σ* is the real part of the eigenvalue), about 15 wingbeat periods; for the lateral unstable mode, *t*_d_ = 81.5 ms, about the same as that of the longitudinal unstable mode.

Now we look at forward flight. First, we consider the longitudinal modes (Table 4). When flight speed is low (*V*_e_ = 1.6 m/s), the modal structure (*λ*_1_ and *λ*_2_ = 0.0307 ± 0.0901*i*; *λ*_3_ = −0.0864; *λ*_4_ = −0.0139) is same as that of hovering flight. At medium speed (*V*_e_ = 3.1 m/s), the modal structure changes to the following: two oscillatory modes, one being nearly neutral (*λ*_1_ and *λ*_2_ = 0.0167 ± 0.0955*i*) and one being stable (*λ*_3_ and *λ*_4_ = −0.0452 ± 0.0109*i*). At higher speeds (*V*_e_ = 4.6–8.6 m/s), the modal structure changes again, having four real modes: the first one is a strongly unstable mode (*λ*_1_, positive and large), the second one is weakly unstable mode (*λ*_2_, positive and small), and the third and fourth ones are stable modes (*λ*_3_ and *λ*_4_, negative). It is important to note that a large change in the stability property occurs when *V*_e_ is larger than about *V*_e_ = 3.1 m/s: *λ*_1_ increases greatly; that is, the instability become much stronger at high speeds. At *V*_e_ = 8.6 m/s, *t*_d_ = 10.1 ms, approximately 8 times smaller than that of the hovering case.

Next we consider the lateral modes (Table 4). In the whole flight-speed range, modal structure does not change: two real modes and one oscillatory mode. The oscillatory mode (*μ*_2_ and *μ*_3_) and one of the real mode (the fast one, *μ*_4_) are always stable. The mode represented by *μ*_1_ changes with speed as following. At hovering (*V*_e_ = 0 m/s) and low flight speed (*V*_e_ = 1.6 m/s), this mode is weakly unstable. At higher speeds (*V*_e_ = 3.1–8.6 m/s), this weakly unstable mode change to a very weakly stable one. The magnitude of *μ*_1_ keeps being very small thus this mode can be treated as a neutral mode. At all speeds, the two stable modes keep being stable, although the magnitude of *μ*_4_ increases greatly and that of the real part of *μ*_2_ (or *μ*_3_) decrease slightly.

The following can be stated from the above analysis. At *V*_e_ = 0 m/s (hover flight), the motion of the dronefly is weakly unstable owing to two weakly unstable natural modes of motion. At forward flight, at low and medium speeds (*V*_e_ = 1.6 m/s and 3.1 m/s), when *V*_e_ increases, the stable modes tend to be more stable, and the unstable modes keep being weakly unstable or approximately neutral. However, at higher speeds (*V*_e_ = 4.6–8.6 m/s), the longitudinal unstable mode, represented by *λ*_1_, becomes strongly unstable, *t*_d_ is 41.2, 16.1 and 10.1 ms for *V*_e_ = 4.6 m/s, 6.9 m/s and 8.6 m/s, respectively.

### Comparison with previous results

As discussed in the Introduction section, the flight stability in forward flight was studied previously using the flight data of a bumblebee. However, the flight data were only for *V*_e_ = 0–4.5 m/s^15^; data at higher flight speeds were not measured. The dronefly in the present study has approximately the same size and same wing kinematics as the bumblebee, and flight data for the whole speed range are available. Therefore, we will make a comparison of the present dronefly results with the previous results of the bumblebee.

The bumblebee’s eigenvalues at various flight speeds^13,14^ are listed in Table 5. It is seen that the variations of the eigenvalue (i.e. the modes of motion) with the flight speed are similar to that of the dronefly. Especially, when *V*_e_ goes from 3.5 m/s to 4.5 m/s, the longitudinal unstable mode (*λ*_1_) becomes much more unstable: at *V*_e_ = 3.5 m/s *t*_d_ = 385 ms, while at *V*_e_ = 4.5 m/s *t*_d_ = 23.5 ms. With present dronefly data, it is shown that for higher flight speeds, the instability become even stronger at *V*_e_ = 6.9 m/s and 8.6 m/s, *t*_d_ become much smaller, *t*_d_ = 16.1 ms and 10.1 ms, respectively. We thus see that the present study extends the flight range of the previous study and provides the stability properties of the full speed range.

**Table 5:** Eigenvalues of bumblebee at various flight speeds^13,14^.

Longitudinal eigenvalues				
*V*_e_ (m/s)	*λ*_1_	*λ*_2_	*λ*_3_	*λ*_4_
0.0	0.045 + 0.129i	0.045–0.129i	−0.197	−0.012
1.0	0.060 + 0.102i	0.060–0.102i	−0.172	−0.023
2.5	0.007 + 0.134i	0.007–0.134i	−0.048 + 0.044i	−0.048–0.044i
3.5	0.012 + 0.120i	0.012–0.120i	0.074 + 0.042i	0.074–0.042i
4.5	0.197	0.049	−0.340	−0.034
**Lateral eigenvalues**				
***V***_**e**_ **(m/s)**	***μ***_**1**_	***μ***_**2**_	***μ***_**3**_	***μ***_**4**_
0.0	0.094	−0.118 + 0.072i	−0.118–0.072i	−0.686
1.0	0.045	−0.088 + 0.076i	−0.088–0.076i	−0.914
2.5	0.000	−0.119 + 0.208i	−0.119–0.208i	−0.971
3.5	−0.006	−0.157 + 0.306i	−0.157–0.306i	−1.088
4.5	−0.014	−0.161 + 0.266i	−0.161–0.266i	−1.375

### Possible effects of the strong instability at high flight speed

As seen above, at the highest flight speed (*V*_e_ = 8.6 m/s), the instability is very strong: the corresponding dimensional eigenvalue is 68.5 s^−1^ and *t*_d_ = 10.1 ms. The wingbeat period *T* = 5.6 ms (*n* = 180 Hz, Table 1). That is, in less than two wingbeats, the magnitude of the disturbance will be doubled; this a very fast growth rate.

Some thoughtful experiments have been done by Ristroph *et al*.^21^ and they succeeded in measuring the control reaction time of fruit-flies. They showed that certain fast sensory organ can give control reaction in about 10–12 ms.

It is reasonable to assume that the dronefly has the same reaction delay time, approximately 11 ms. We thus see that at the highest speed, the time for the disturbance to double its magnitude (*t*_d_ = 10.1 ms) is approximately the same as the sensory reaction time of the fly. This indicates that the flight control is already difficult for flight at this speed. If flight speed is further increased, the instability may become even stronger; then the fly may not be able to control the flight. Therefore, we may say that strong flight instability may be one of the factors that limit the flight speed of a fly. Other factors could be very larger power requirement and inability to produce enough lift or thrust.

## Conclusions

The longitudinal derivatives due to the lateral motion are approximately 3 orders of magnitude smaller than other longitudinal derivatives; thus, one can decouple the longitudinal motion from the lateral motion, as people do for a conventional airplane. At hovering flight, the motion of the dronefly is weakly unstable owing to two unstable natural modes of motion, a longitudinal one and a lateral one. At low (1.6 m/s) and medium (3.1 m/s) flight speeds, the unstable modes become even weaker and the flight is approximately neutral. At high flight speeds (4.6 m/s, 6.9 m/s and 8.6 m/s), the flight becomes more and more unstable due to an unstable longitudinal mode. At the highest flight speed, 8.6 m/s, the instability is so strong that the time constant representing the growth rate of the instability (disturbance doubling time) is only 10.1 ms, which is close to the sensory reaction time of a fly, approximately 11 ms. This indicates that strong instability may play a role in limiting the flight speed of the insect.

## Supplementary information

Supplementary Information.
