# Supplementary material for: Forward flight stability in a drone-fly
Source: Sci Rep. 2020 Feb 6;10:1975. doi: 10.1038/s41598-020-58762-5 (PMC7005165; doi:10.1038/s41598-020-58762-5)
Supplement: Supplementary file 1 — Supplementary Information. [file 41598_2020_58762_MOESM1_ESM.pdf]

Electronic Supplemental Material to "Forward flight stability in a drone-fly"

Hao Jie Zhu[1,*], Xue Guang Meng[2], and Mao Sun[1]

[1]Ministry-of-Education Key Laboratory of Fluid Mechanics, School of Aeronautic and Engineering, Beihang University, Beijing, 100191, China

[2]State Key Laboratory for Strength and Vibration of Mechanical Structures, School of Aerospace, Xi'an Jiaotong University, Xi'an, 710049, China

*by1505144@buaa.edu.cn

S1 The flow solution method and evaluation of aerodynamic forces and moments

The flow equations (the Navier-Stokes equations) and the solution method used in the present study are the same as those described in Sun and Tang[1] and Sun and Xiong[2]. Once the flow equations are numerically solved, the fluid velocity components and pressure at discretized grid points for each time step are available. The aerodynamic forces and moments acting on the wing (or the body) are calculated from the pressure and the viscous stress on the wing (or the body) surface. Resolving resultant aerodynamic force of the wing into the *z*- and *x*-axes, we obtain the vertical ($-z^{+}_{\mathrm{e,w}}$) and the horizontal ($x^{+}_{\mathrm{e,w}}$) forces of the wing, respectively. Let $m^{+}_{\mathrm{y_1,w}}$ be the moment about the y_1-axis (which passes the wing root). The pitching moment about the center of mass of the insect due to the aerodynamic force of the wing ($m^{+}_{\mathrm{e,w}}$) can be calculated as (see Ref. 3):

$$m^{+}_{\mathrm{e,w}} = m^{+}_{\mathrm{y_1,w}} + x^{+}_{\mathrm{e,w}} \cdot l_1 \cos(\chi_0 - \chi) / c - x^{+}_{\mathrm{e,w}} \cdot l_1 \sin(\chi_0 - \chi) / c \qquad (1)$$

S2 Code validation and grid resolution test

The flow equations were solved over moving overset grids because there are relative movements between the left and right wings and between the body and wing. There was a body-fitted curvilinear grid for each of the wings and the body and a background Cartesian grid which extends to the far-field boundary of the domain (Fig. S1). In the calculation process, the wing grid was regenerated in every time step because of the spanwise-twist deformation. The code was tested in Wu and Sun[4] using experimental data on a revolving model bumblebee wing and recently in Cheng and Sun[5] using measured aerodynamic

forces on a flapping model fruit fly wing. The measured and computed results were in good agreement.

Before proceeding to compute the flows, grid resolution tests were conducted to ensure that the flow calculation was grid independent. In the tests, the wing kinematics at the highest flight speed (8.6 m/s) was taken and three grid systems were considered. For grid system 1, the wing grid had dimensions 41 × 61 × 45 in the normal direction, around the wing, and in the spanwise direction, respectively (first layer grid thickness was 0.0015*c*); the body grid had dimensions 41 × 81 × 45 along the body, in the azimuthal direction and in the normal direction, respectively; the background grid had dimensions 88 × 79 × 79 in the x, y and z directions, respectively. For grid system 2, the corresponding grid dimensions were 61 × 91 × 69, 61 × 121 × 67 and 130 × 117 × 117 (0.001*c*). For grid system 3, the corresponding grid dimensions were 91 × 135 × 101, 91 × 181 × 99 and 193 × 173 × 173 (0.00067*c*). For all three grid systems, the outer boundary of the background grid was 30*c* from the wing root in *y* and *z* direction, and 60*c* in *x* direction as extended in the downstream field; the grid points of the background grid concentrated in the near field of the wings where its grid density was approximately the same as that of the outer part of the body grid. The non-dimensional aerodynamic forces computed by three grid systems are shown in Fig. S2. As oberserved from Fig. S2, the first grid refinement produces small difference in aerodynamic forces and the second grid refinement produces almost no difference. Calculations were also conducted using a larger computational domain by adding more grid points to the outside of the background grid of grid system 3 (the outer boundary was increased to 50*c* in *y* and *z* direction, 80*c* in *x* direction). The calculated results showed that there was no need to put the outer boundary further than that of grid system 3. The non-dimensional time step was 0.02 (non-dimensionalized by *c/U*). The effect of time step value was studied and it was found that a numerical solution effectively independent of the time step was achieved if the time step value was 0.02. From the above discussion, it was concluded that grid system 3 and time step 0.02 were appropriate for the calculation.

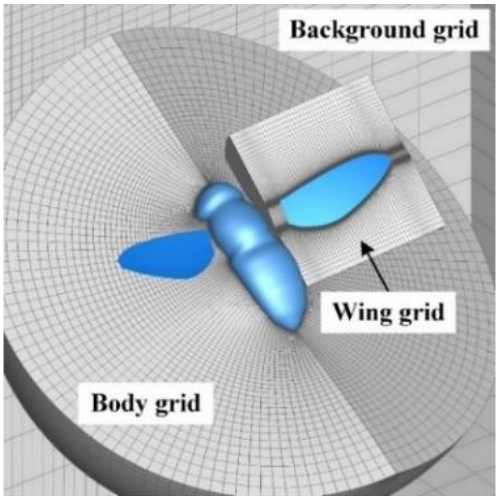

Figure S1. Model insect and portions of computational grids.

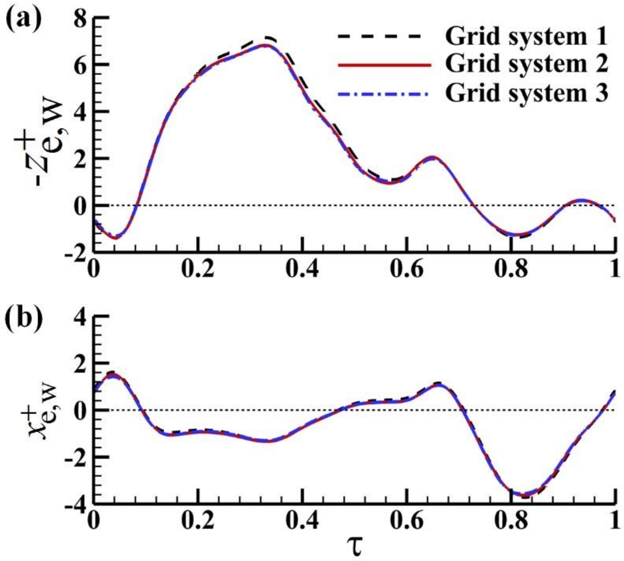

Figure S2. Time courses of non-dimensional aerodynamic forces in a flapping cycle calculated by three grid systems.

Supplementary References

1. Sun, M. & Tang, J. Unsteady aerodynamic force generation by a model fruit fly wing in flapping motion. *J. Exp. Biol.* **205**, 55–70 (2002).
2. Sun, M. & Xiong, Y. Dynamic flight stability of a hovering bumblebee. *J. Exp. Biol.* **208**, 447–459 (2005).
3. Ellington, C. P. The aerodynamics of hovering insect flight. II. Morphological parameters. *Philos. Trans. R. Soc. London B Biol. Sci.* **305**, 17–40 (1984).
4. Wu, J. H. & Sun, M. Unsteady aerodynamic forces and power requirements of a bumblebee in forward flight. *Acta Mech. Sin.* **21**, 207–217 (2005).
5. Cheng, X. & Sun, M. Very small insects use novel wing flapping and drag principle to generate the weight-supporting vertical force. *J. Fluid Mech.* **855**, 646–670 (2018).
